# Solid-state intramolecular motions in continuous fibers driven by ambient humidity for fluorescent sensors

**DOI:** 10.1093/nsr/nwaa135

**Published:** 2020-06-17

**Authors:** Yunmeng Jiang, Yanhua Cheng, Shunjie Liu, Haoke Zhang, Xiaoyan Zheng, Ming Chen, Michidmaa Khorloo, Hengxue Xiang, Ben Zhong Tang, Meifang Zhu

**Affiliations:** State Key Laboratory for Modification of Chemical Fibers and Polymer Materials, International Joint Laboratory for Advanced Fiber and Low-Dimension Materials, College of Materials Science and Engineering, Donghua University, Shanghai 201620, China; State Key Laboratory for Modification of Chemical Fibers and Polymer Materials, International Joint Laboratory for Advanced Fiber and Low-Dimension Materials, College of Materials Science and Engineering, Donghua University, Shanghai 201620, China; Department of Chemistry, the Hong Kong Branch of Chinese National Engineering Research Center for Tissue Restoration and Reconstruction, Institute for Advanced Study, and Department of Chemical and Biological Engineering, Hong Kong University of Science and Technology, Hong Kong, China; Department of Chemistry, the Hong Kong Branch of Chinese National Engineering Research Center for Tissue Restoration and Reconstruction, Institute for Advanced Study, and Department of Chemical and Biological Engineering, Hong Kong University of Science and Technology, Hong Kong, China; Beijing Key Laboratory of Photoelectronic/ Electrophotonic Conversion Materials, Key Laboratory of Cluster Science of Ministry of Education, School of Chemistry and Chemical Engineering, Beijing Institute of Technology, Beijing 100081, China; Department of Chemistry, the Hong Kong Branch of Chinese National Engineering Research Center for Tissue Restoration and Reconstruction, Institute for Advanced Study, and Department of Chemical and Biological Engineering, Hong Kong University of Science and Technology, Hong Kong, China; Department of Chemistry, the Hong Kong Branch of Chinese National Engineering Research Center for Tissue Restoration and Reconstruction, Institute for Advanced Study, and Department of Chemical and Biological Engineering, Hong Kong University of Science and Technology, Hong Kong, China; State Key Laboratory for Modification of Chemical Fibers and Polymer Materials, International Joint Laboratory for Advanced Fiber and Low-Dimension Materials, College of Materials Science and Engineering, Donghua University, Shanghai 201620, China; Department of Chemistry, the Hong Kong Branch of Chinese National Engineering Research Center for Tissue Restoration and Reconstruction, Institute for Advanced Study, and Department of Chemical and Biological Engineering, Hong Kong University of Science and Technology, Hong Kong, China; State Key Laboratory for Modification of Chemical Fibers and Polymer Materials, International Joint Laboratory for Advanced Fiber and Low-Dimension Materials, College of Materials Science and Engineering, Donghua University, Shanghai 201620, China

**Keywords:** solid-state molecular motion, fibers, aggregation-induced emission, humidity sensors

## Abstract

One striking feature of molecular rotors is their ability to change conformation with detectable optical signals through molecular motion when stimulated. However, due to the strong intermolecular interactions, synthetic molecular rotors have often relied on fluid environments. Here, we take advantage of the solid-state intramolecular motion of aggregation-induced emission (AIE) molecular rotors and one-dimensional fibers, developing highly sensitive optical fiber sensors that respond to ambient humidity rapidly and reversibly with observable chromatic fluorescence change. Moisture environments induce the swelling of the polymer fibers, activating intramolecular motions of AIE molecules to result in red-shifted fluorescence and linear response to ambient humidity. In this case, polymer fiber provides a process-friendly architecture and a physically tunable medium for the embedded AIE molecules to manipulate their fluorescence response characteristics. Assembly of sensor fibers could be built into hierarchical structures, which are adaptive to diverse-configuration for spatial-temporal humidity mapping, and suitable for device integration to build light-emitting sensors as well as touchless positioning interfaces for intelligence systems.

## Teaser

Highly sensitive optical fiber sensors were developed by combining the advantages of both the solid-state intramolecular motion of AIE molecular rotors and one-dimensional fibers, which can respond to ambient humidity with observable fluorescence color change in a rapid and reversible fashion.

## INTRODUCTION

Molecular motion is one of the intrinsic characteristics of matter; nature utilizes molecular-scale motors to generate biological processes and power sophisticated life behaviors [1,2]. The development of synthetic molecular motors that are stimuli-responsive and capable of converting an input energy into macroscopic signals provide fascinating prospects for smart materials to mimic the ingenious functions of life [3,4]. Drawing lessons from nature, a number of molecular motors have been developed with precisely controlled translational and rotary motions, where reversible reorganization of the molecular conformation is allowed on exposure to a stimulus [3,5]. Recent studies introduced functional groups into molecular motors to generate electrochemical or fluorescence output signals, translating the dynamic molecular-scale motions into detectable macroscopic signals [6,7]. However, they are mostly exploited in gas or liquid state. Seldom are examples investigated in engineering solid forms because of the strong intermolecular interactions [8]. If the molecular motions are able to be maintained in the solid state and endowed with optical responses, they will ultimately generate a breakthrough in stimuli-responsive materials for fast detection and identification based on colorimetric or fluorescent changes.

In order to meet the above criteria, fluorescent molecular systems with aggregation-induced emission (AIE) phenomenon hold great potential [9,10]. AIE-active molecules are characterized by the highly twisted propeller-like conformation [11], which shows non-luminescence or weak luminescence in isolated state but exhibits intense luminescence in aggregates because of restricted intramolecular motion (RIM) [12,13]. The RIM working mechanism of AIE molecular systems prevents the dissipation of excited-state energy through non-radiative relaxation process, strengthening the fluorescence in aggregate [14–16]. Through intramolecular motion, these AIE-based fluorescent molecules with high conformational flexibility show remarkable changes in luminescence upon reaction to different surrounding microenvironment conditions (e.g. temperature [17,18], viscosity [19], pressure [20], light [21] and rigidity [22,23]). Moreover, the weak intermolecular interactions between twisted AIE molecules endow them with strong motion capability, facilitating spontaneous conformation changes even in the crystalline state [24], making it possible for them to work in engineering solid state [8,25,26].

Water vapor widely exists everywhere; the sensitive sensing is important in controlling industrial systems and sustaining living organisms. Molecular rotors characterized with conjugated electron-donor (D)-acceptor (A) structure is of particular interest for water molecule diagnosis because of the twisted intramolecular charge-transfer (TICT) effect [27,28]. Their emissions red-shift and quench when the local environment polarity increases [29]. TICT effect is driven by the intramolecular rotation of D-A subgroups [30–32]. Combining the effect of AIE and TICT manipulated by intramolecular motions, a sensitive humidity sensor could be developed by embedding AIE molecules into a water-captured polymer. In addition, sensor materials fabricated from scalable processes that are mechanically compliant when bent and folded are also favorable for the future integrated system.

We now take advantage of intramolecular motion of D-A based AIE molecules and 1D polymer fibers to develop a smart AIE-based humidity sensor. Upon adsorption of water vapor, the swelled polymer fiber triggers the binary effect of AIE and TICT via the regulation of intramolecular motions, generating different fluorescence color to quantify and map variations of local humidity. Moreover, micro-/nano-fibrous structure from continuous solution-spinning leads to beneficial properties of mechanical flexibility, high surface-to-volume ratio and facile scalable fabrication, accounting for high sensitivity and fast response to ambient humidity. In particular, assembly of sensor fibers could form hierarchical structures (coils, meshes, fabrics), which are adaptive to diverse-configuration and suitable for integration into flexible devices and artificial intelligence systems.

## RESULTS AND DISCUSSION

### Intramolecular motions of AIE molecules

**Figure 1.**
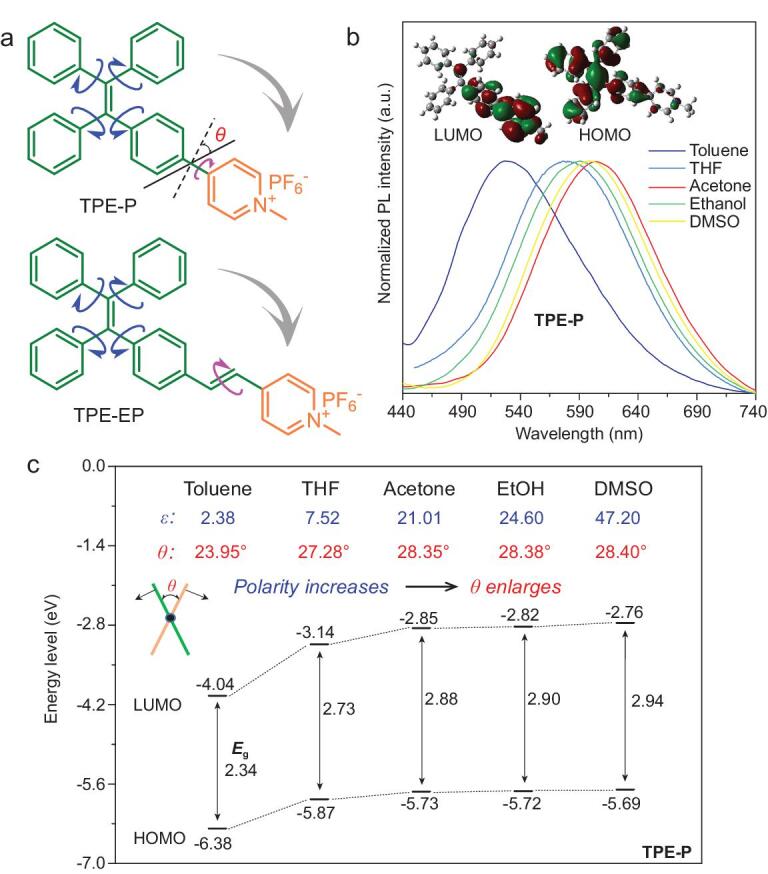
Molecular structures and their intramolecular motion capability. (a) Chemical structures of TPE-P and TPE-EP. (b) Normalized fluorescence spectra of TPE-P in various organic solvents. Inset: the representative molecular orbital distributions (left: LUMO; right: HOMO) of TPE-P in toluene solvent (ϵ = 2.38). (c) Calculated ground state HOMO-LUMO energy profiles of TPE-P and its θ between pyridinium group and TPE unit in various solvents. DFT calculations are performed with B3LYP/6–31G(d) using Gaussian 09 program.

In this study, D-A based AIE salt molecules are used as reporters and hydrophilic commercial polymers are applied as a water-captured network. A scalable solution-spinning process is followed to fabricate continuous AIE/polymer fibers. Specifically, D-A based AIE molecules of TPE-P [33] and TPE-EP [34] were utilized to exhibit humidity-responsive properties (Fig. 1a). They contain three segments: an electron-donating tetraphenylethene (TPE) group, an electron-accepting pyridinium salt unit, and a spacer unit of single (TPE-P)/double (TPE-EP) bond. The highly twisted TPE group with four phenyl rings ensures the intramolecular motion ability in solid state, providing the structure with the flexibility to respond to the surrounding environment (Supplementary Fig. 1 and Table 1) [33]. Meanwhile, the pyridinium salt unit imparts strong D-A interaction to form TICT state under polar environment (e.g. water), which can be demonstrated by a density functional theory (DFT) calculation. The highest occupied molecular orbital (HOMO) is mainly localized on the TPE group, whereas the lowest unoccupied molecular orbital (LUMO) is more concentrated on the pyridinium unit (inset, Fig. 1b). On the other hand, the pyridinium salt group also improves the compatibility between AIE molecules and the hydrophilic polymer matrix.

In solution, TPE-P exhibits a notable solvatochromic luminescence shift from blue to orange when the solvent polarity changes from *ϵ* (dielectric constant) = 2.38 in toluene (*λ*_em_ = 527 nm, green, *Ф*_F_ = 2.8%) to *ϵ =* 47.20 in dimethyl sulfoxide (DMSO, *λ*_em_ = 599 nm, orange, *Ф*_F_ = 0.4%) (Fig. 1b). Meanwhile, the absorption spectra of TPE-P blue-shifted with increasing solvent polarity (Supplementary Fig. 2), which is consistent with the calculated energy gap (Δ*E*_g_) in Fig. 1c. These results indicate the excellent TICT property of the D-A based AIE molecules. As expected, the rotation angle (*θ*) between D-A subgroups of TPE-P enlarges from 23.95° to 28.40° with an increasing number of *ϵ* at the ground state (inset, Fig. 1c). In addition, when introducing double bond in between TPE and pyridinium groups, the redder photoemission of TPE-EP was observed to compare to those of TPE-P because of the enhanced conjugation (Supplementary Fig. 3), suggesting the possibility of chemical modification to achieve the desired emission range. Therefore, in the condensed state, the emission properties of AIE molecules can also be sensitive to the environmental polarity provided by the presence of the highly propeller-like twisted structure of TPE group.

### Fiber sensor design and mechanism

**Scheme 1.**
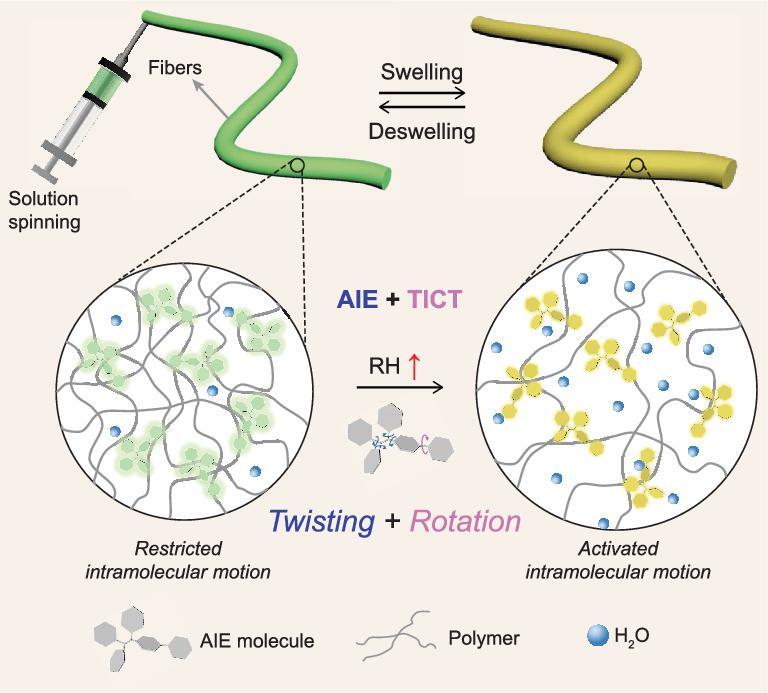
Schematic illustration of fluorescence variation of AIE/polymer fiber sensor when exposed to water molecules. The adsorption of water molecules into hydrophilic polymers causes the swelling of the fibers and increases the polarity of the local microenvironment, which promotes the excited-state intramolecular twisting (phenyl rings in TPE unit) and ground-state rotation (D-A subgroups) of AIE molecules and results in the red-shifted and decreased fluorescence. The recovery of fluorescence can be achieved by the desorption of water molecules from polymer fibers.

The mechanism for humidity sensing is intramolecular motion-induced variation of fluorescent color and intensity (Scheme 1). In the dry state, the rigid polymer matrix with a low content of water molecules (low polarity, low relative humidity (RH)) restricts intramolecular motions of AIE molecules, resulting in emissions with short wavelength and high intensity. In the swollen state, the soft polymer matrix adsorbed with ambient water molecules (high polarity, high RH) has more freedom to facilitate the intramolecular twisting of the phenyl rings within the TPE unit upon excitation. The activated intramolecular motions play as a non-radiative channel to decay the energy of the excited state, resulting in the weak fluorescence emission. Meanwhile, the water-adsorbed polymer matrix with high polar environment promotes the intramolecular rotation between the TPE unit and pyridinium group to form TICT state. These factors together activate the intramolecular motions, resulting in red-shifted and weakened emission of AIE molecules as humidity rises [35]. Furthermore, the small diameter of the fibers results in a high surface area that can be easily accessed by the water molecules, enabling water molecules to diffuse in and out of the fibers rapidly [36,37]. Reversible adsorption and desorption of water molecules within fibers powers the dynamic intramolecular motions of AIE molecules, showing high sensitivity to ambient humidity with obvious fluorescence variations.

### Continuous AIE/polymer microfibers by dry-spinning

**Figure 2.**
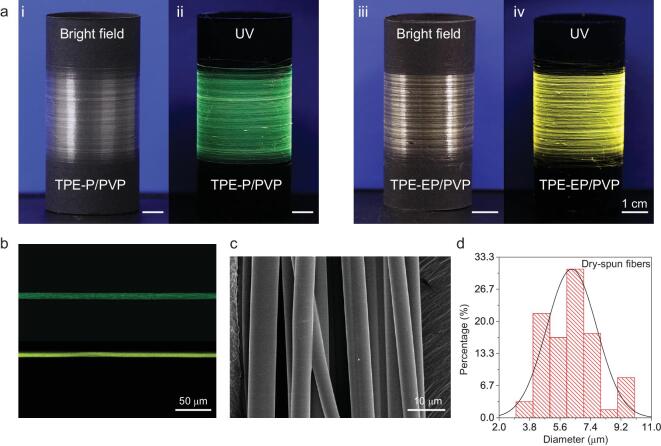
1D AIE/PVP micro-fibers. (a) Photographs of continuous dry-spun AIE/polymer microfibers wound on black-paper coated drums. (Left) TPE-P/PVP (at ambient humidity: 38%RH) and (right) TPE-EP/PVP (at ambient humidity: 47%RH) fibers taken under (i and iii) daylight and (ii and iv) 365 nm UV light. (b) Microscopic confocal representation of the AIE/PVP fibers with homogeneous fluorescence distribution. For TPE-P/PVP, *λ*_ex_ = 380 nm; for TPE-EP/PVP, *λ*_ex_ = 410 nm. (c) SEM image of the as-prepared microfibers (TPE-P/PVP) with smooth surface and straight interface. (d) Fiber diameter distribution of dry-spun fibers.

In nature, silks with the diameters of several micrometers are produced from spiders or silkworms by directly spinning a silk protein dope through a spinneret. The solidification into a fiber is initiated immediately once pulled out [38,39]. Inspired by this natural spinning method, anisotropic dry-spinning technologies have shown advantages of easy operation and low cost, which is achieved by the evaporation of a volatile solvent to solidify the fiber during the spinning process (Supplementary Fig. 4). To fabricate AIE/polymer microfibers, dry-spinning technology was utilized. Considering the solution viscosity, materials processability, and water absorptivity, polyvinylpyrrolidone (PVP) was chosen as a material support for microfiber fabrication. We directly collected the highly uniform fibers by continuous extrusion of AIE/PVP ethanol solution under a desired drawing speed. Figure 2a presents continuous and transparent TPE-P/PVP and TPE-EP/PVP fibers at the ambient environment, which show strong green and yellow fluorescence under UV excitation, respectively. The confocal microscopic images of the individual fibers illustrate the homogeneous dispersion of AIE molecules within the fibrous PVP network (Fig. 2b). Such a 1D fibrous structure was further studied by the scanning electron microscope (SEM), exhibiting a smooth surface and straight boundary with a mean diameter of ∼6 μm (Fig. 2c and d, Supplementary Fig. 5).

### Smart humidity sensing of fiber sensor

**Figure 3.**
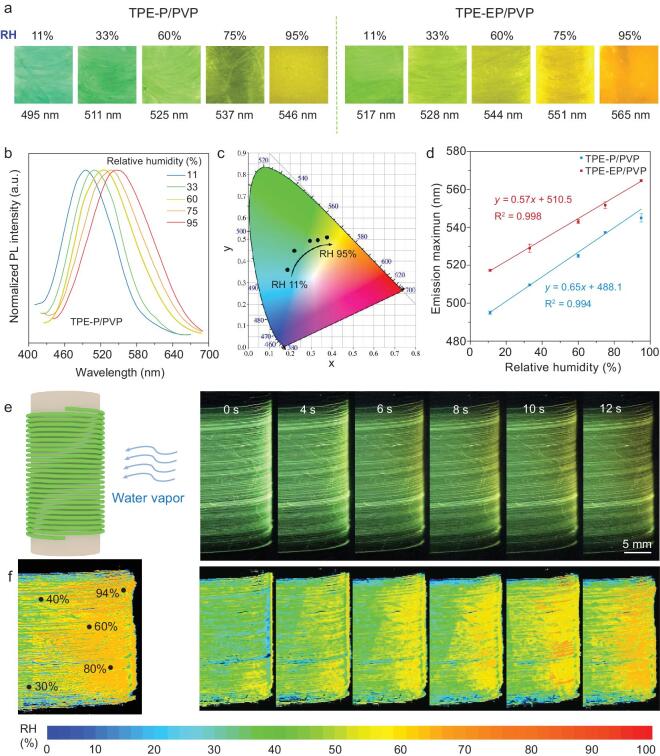
Sensor response characteristics of AIE/PVP microfibers. (a) Fluorescent images of TPE-P/PVP (left) and TPE-EP/PVP (right) microfibers at different RH under UV irradiation. (b) Normalized PL spectra of TPE-P/PVP microfiber sensors and (c) corresponding CIE 1931 coordinates in a standard color space at various RH. *λ*_ex_ = 375 nm. (d) Plots of emission maximum versus the RH with linear relationships. (e) The schematic illustration of the AIE/PVP fibers exposed to moisture (left). Lateral photographs of the fluorescence process within the TPE-P/PVP fibrous film taken under 365 nm UV light (right). The photos are video frames captured from Video 1 (online Supplementary Data) at a different time. The ambient RH is around 27%. (f) The corresponding humidity gradient maps calculated by a MATLAB program, showing the moisture diffusion process. The calculated RH values of the chosen places presented on the humidity map.

The dry-spun microfibers containing microenvironment-responsive AIE molecules can be considered for an optical humidity sensor. Fluorescent emission photographs of AIE/PVP microfibers after exposure to humid environments are shown in Fig. 3a. Upon increasing RH from 11% to 95%, the results show the TPE-P/PVP fibrous film exterts an obvious fluorescence color change from blue to yellow, whereas the emission of TPE-EP/PVP changes from green to orange correspondingly. We used photoluminescence (PL) spectroscopy to give the normalized PL spectra of AIE/PVP at different RH. The emission of TPE-P/PVP gradually red-shifted from 495 nm to 546 nm (Fig. 3b), while TPE-EP/PVP also turned from 517 nm to 565 nm (Supplementary Fig. 6a) progressively. Such a remarkable red-shifted emission revealed that AIE molecules featured an obvious TICT property. The materials sensitivity was reversible by varying the ambient moisture alternately (Supplementary Fig. 7). The corresponding chromaticity diagram illustrates quantitatively the range of fluorescence colors (Fig. 3c, Supplementary Fig. 6b). Linear relationships (R^2^ > 0.99) were obtained for the plot of the emission maximum vs. the RH (Fig. 3d), indicating the capability of AIE/PVP fibrous materials for quantitative calibration of environmental humidity.

Benefitting from the high humidity responsiveness of AIE/PVP fibrous materials, the spatial-temporal mapping of moisture distribution became possible based on the correlation between the RH information and optical signal. The evolution of humidity distribution was monitored by exposing one side of TPE-P/PVP fibrous film to moisture (Fig. 3e, Supplementary Video 1). Persistent moisture flow from a humidifier leads to a high RH at the right side and low RH at the left side (Fig. 3e). The moisture gradient was introduced into the fibrous film to cause the emission response. The emission variations were observed and tracked, and a moisture diffusion pathway within the fibrous materials could be simply investigated. To quantitatively translate fluorescence signals into humidity information, the emission spectra of TPE-P/PVP at various RH between 11% and 95% were collected, and then converted into the CIE coordinates. It is found that the extracted chromaticity coordinates exhibit a linear relationship between RH, resulting in one-to-one correspondence (Supplementary Fig. 8). As a consequence, the spatial-temporal fluorescence mapping with a resolution of  ∼60 μm was converted into a humidity gradient distribution (Fig. 3f), resulting in real-time measurement of local humidity within fibrous materials. In addition, the emission intensity decreases drastically accompanied by the increased RH (Supplementary Fig. 9), which is a result of the activated intramolecular motions within the softened and polar polymer network.

### Textile displays of microfiber sensor

**Figure 4.**
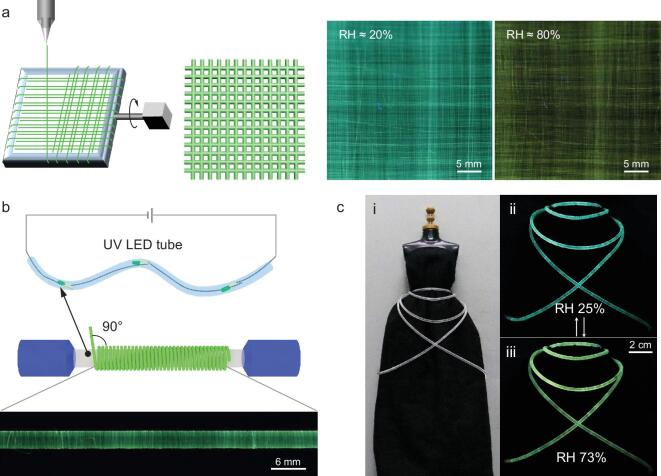
Textile displays of microfiber sensor. (a) Schematic illustration for weaving the AIE/PVP fibrous textile (left). Fluorescent photos of the microfiber textile taken at RH of 20% and 80%, respectively (right). (b) Schematic of wrapping a continuous fiber around a silicone UV LED light tube (top) to develop a fiber-shaped humidity device. Fluorescent side-view image of aligned fiber wrapped around the silicone tube with an angle of 90° (bottom). (c) Fiber-shaped device integrated with an apparel dress (i). The images of the sensor fabric taken at RH of 25% (ii) and 73% (iii), respectively.

The continuous fiber spinning process enables production of fiber textile, which was achieved by aligning fiber arrays on a Teflon frame at alternately parallel and perpendicular directions (Fig. 4a, Supplementary Fig. 10). The integrated textile shows aligned morphology. The chromatic emission transition from blue-green to yellow of TPE/PVP fabric suggests the rise of RH, corresponding to the conditions of the surrounding environment. Similar to a chameleon, our AIE-based smart fabrics are able to change the emission color in response to external humidity, and are able to adapt to arbitrary surfaces for various application scenarios. Integration of these smart fibers with apparels is of great interest in wearable systems [40]. Figure 4b shows the fabrication process for the integration of a fiber-shaped device. A silicone tube was first inserted with tandem-connected UV LED light beads, then the dry-spun microfibers were uniformly wrapped around the modified silicone tube to produce the designed fiber-shaped device. Such fabrication process was synchronous with the dry-spinning, and is suitable for large-scale production. The obtained fiber-shaped device was then integrated with the apparel dress as shown in Fig. 4c(i). When linked to the external circuit, the light color automatically changed from green (ii) to yellow (iii) upon RH increases that were readily observed with the naked eye. The fiber-shaped device can serve as a built-in sensor for easy identification of the ambient humidity and is also able to act as color-tunable lighting for smart displays. Our microfiber-based sensors are flexible and soft, and they can be woven into textiles for highly-integrated wearable systems.

### Fluorescence response of AIE molecules in different polymer matrices

The polymer serves as a water-captured network to provide a microenvironment for AIE molecules when exposed to ambient moisture, and thus allows them to take intramolecular motions to generate different fluorescence for humidity sensing. To understand the correlation between the emission color of the embedded AIE molecules and physicochemical properties of matrix polymers, three other hydrophilic polymers including polyacrylic acid (PAA), sodium polystyrene sulfonate (NaPSS) and polyvinyl alcohol (PVA) were also studied to manipulate the humidity-response performance of AIE molecules.

**Figure 5.**
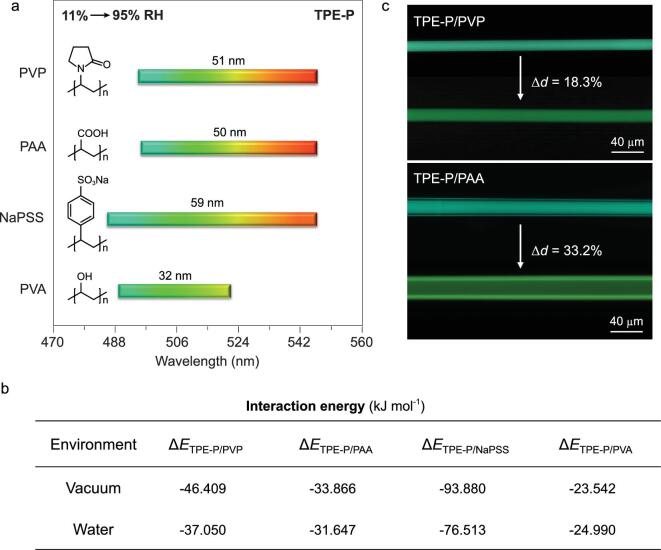
Fluorescence response of TEP-P in different polymers under various RH. (a) Emission maximum shifts of TPE-P in PVP, PAA, NaPSS and PVA from RH of 11% to 95%. (b) Calculated interaction energies of TPE-P/polymers in vacuum and water. To simplify the DFT calculation, vacuum and water were used to stand for the environments of low RH and high RH, respectively. (c) Microscopic fluorescent images of TPE-P/PVP and TPE-P/PAA microfibers before (20%RH) and after (70%RH) exposure to water vapor.

Figure 5a shows the effects of polymer chain flexibility on responsiveness of AIE molecules. As RH increases, emission of AIE/polymers red-shift accordingly (Supplementary Figs 11 and 12). At low RH of 11%, TPE-P-doped NaPSS exhibits a shorter wavelength than PVP and PAA. In NaPSS, abundant intermolecular interactions exist between phenyl groups of NaPSS and AIE molecules to suppress the TICT state formation, in favor of the bluer emission [41]. Such strong interaction was proved by interaction energy (Δ*E*) of TPE-P and polymers from DFT calculations (Fig. 5b, Supplementary Fig. 13) [42]. The Δ*E*_TPE-P/NaPSS_, which is −93.880 kJ mol^−1^ (low RH), is the largest interaction energy among TPE-P and other polymers. At high RH of 95%, PAA and NaPSS matrices embedded with TPE-P show a similar wavelength emission to that of PVP (Fig. 5a), which is determined by their hydrophilic chemical structure. At high RH, water-adsorbed polymers provide a soft network to allow AIE molecules to rotate freely to stabilize the TICT state with longer-wavelength emission, which was further demonstrated by the smaller interaction energies for TPE-P/polymers at high RH than those at low RH (Fig. 5b). Compared with those of TPE-P-embedded amorphous polymers (PVP, PAA and NaPSS), the emission of TPE-P/PVA shows the smallest red-shift range of 32 nm (Fig. 5a), which is due to the existence of crystalline domain in PVA (Supplementary Fig. 14). At low RH, rigid crystalline PVA domain restricts the formation of TICT state of TPE-P to give short-wavelength emission; at high RH, the crystalline PVA phase blocks the water permeation into the polymer matrix to bring about hysteresis in humidity response.

Fast water harvesting of polymers from the ambient environment leads to abrupt intramolecular motions of AIE molecules, resulting in instant response to ambient moisture. TPE-P/PVP and TPE-P/PAA share similar chromatic shift range but exhibit different response time. Upon exposure to prompt high RH, the polymer microfibers swell immediately because of the adsorption of water vapor (Fig. 5c). Within the same time period, inspection of microfibers of TPE-P/PVP and TPE-P/PAA using a fluorescence microscope showed that they swell with diameter enlargement of 18.3% and 33.2%, respectively. As a result, the TPE-P/PAA shows a redder emission compared with that of TPE-P/PVP, indicating faster humidity response of TPE-P/PAA. These results demonstrate that the fluorescence response of AIE molecules can be manipulated by choosing the appropriate polymer matrix.

### Instant fluorescence response of nanofiber sensor

**Figure 6.**
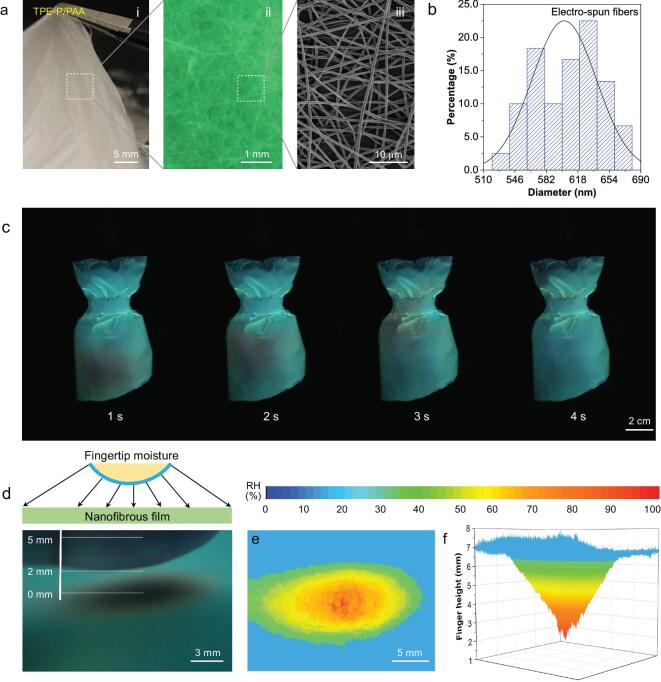
Instant humidity response of TPE-P/PVP nanofibers. (a) A thin nanofibrous electro-spun TPE-P/PVP fabric observed under (i) visible light, (ii) fluorescent microscope and (iii) SEM. The nanofibrous fabric has a typical thickness of few nanometers. (b) A mean diameter of 600 nm of electro-spun TPE-P/PAA nanofibers observed from SEM image. (c) Fluorescent photographs showing the capability of the TPE-P/PAA nanofiber-based dresses to track water vapor flow movement. Water vapor was guided by a plastic tube. The images are video frames from Video 2 (online Supplementary Data) taken at 1, 2, 3 and 4 s, demonstrating the immediate response and fast recovery of TPE-P in nanofibers. (d) Schematic illustration of the lateral position of a fingertip surface above a nanofibrous surface (top). Side-viewed fluorescence mapping of the TPE-P/PAA fabric with an approaching finger (bottom, Video 3, online Supplementary Data). The distances between fingertip and fibrous surface are labeled. (e) Top-viewed 2D signal RH distribution measured by the AIE-based nanofibrous sensor matrix. The transformation of fluorescence signal into RH is calculated by a MATLAB program. (f) Side-viewed 3D mapping of the relative positions of the applied fingertip.

Nanofibers from electro-spinning [43], characterized with large surface area, high porosity and fine flexibility, hold great promise as a physical medium for AIE molecules to achieve instant humidity response sensitivity. Considering fast water adsorption and good processability of PAA network, AIE molecules were incorporated into PAA for instant humidity sensing. Using electro-spinning technique (Supplementary Fig. 15), a light and free-standing nonwoven nanofibrous fabric made of AIE/PAA (thickness: ∼6 μm, Supplementary Fig. 16) was manufactured (TPE-P/PAA, Fig. 6a(i)). The images of fluorescence microscopy (Fig. 6a(ii)) and SEM (Fig. 6a(iii)) confirm that the homogeneous, smooth and continuous nanofibers are developed with an average diameter of 600 nm (Fig. 6b, Supplementary Fig. 17). It is possible to manufacture the fluorescent nanofibrous membrane into diverse architectures (Supplementary Fig. 18). For example, dresses were made using the non-woven textiles (TPE-P/PAA) as shown in Fig. 6c. Compared with microfiber sensor, AIE/PAA nanofibrous membrane show much faster response when exposed to water vapor (Supplementary Fig. 19), which is attributed to its nanostructure and excellent water adsorption of PAA matrix. The fluorescence color changed and recovered immediately (<1 s) upon moving water vapor, providing a potential material in both smart sensing and fashion display (Fig. 6c, Supplementary Fig. 20 and Video 2). Such fast sensitivity is much higher compared with other fluorescent humidity-sensitive materials [36,44,45], and even comparable with those of carbon-based electronic devices [46,47].

Owing to the water secretion from sweat pores of fingers [48], humid atmosphere is generated around the near surface of fingertips (Fig. 6d, top). The sensitive response of AIE/PAA nanofibrous membrane can be demonstrated by the touchless sensing of a human finger (Fig. 6d, bottom). When the finger approached the TPE-P/PAA nanofibrous fabric, the green emissive fabric instantaneously turned into dark orange with apparent finger outline (Supplementary Video 3). Notably, the water molecules that induce the response of the embedded TPE-P in nanofibers are secreted from a negligible amount of water on the fingertips [48], demonstrating the abrupt intramolecular motions of AIE molecules during this process. Based on the correlation between the RH and fluorescence color, the moisture distribution around the fingertip was displayed. Each pixel in Fig. 6e was transformed into the individual RH using MATLAB program (Supplementary Fig. 21). After calibration of RH versus distance from the fingertip (Supplementary Fig. 22), a reconstructed image with spatial humidity distribution signals coincided with the real position of the fingertip (Fig. 6f). That is, our AIE-based nanofibrous fabrics can serve as touchless positioning interface for intelligent human-machine interfaces. Such smart optical materials have no need for electronic assembly [47,49], providing an alternative strategy for future integrated wearable systems.

## CONCLUSION

In summary, we prepared AIE/polymer fibers as a highly sensitive fluorescence humidity sensor, which adopts the mechanism of intramolecular motion of AIE molecular rotors based on AIE and TICT effects. Upon water physisorption of polymer fibers, the embedded AIE molecules reversibly change color and show linear response to the relative humidity in the wide range of 11% to 95%. Such fluorescence response performance could be amplified by refining the fiber structure and changing the chemical structure of polymers, which enables water molecules to diffuse in and out of the fibers rapidly. In addition, fibrous structure sensors can be used to build various architectures, facilitating multifunctionality in terms of spatial humidity mapping, high device-integration capability and touchless positioning. We expect our strategy of combining AIE and 1D fiber structure will not only provide a new route for humidity sensor, but also serve as artificial nerves to sense wide environmental stimuli.

## METHODS

### Materials

AIE molecules of TPE-P and TPE-EP were synthesized according to the literature method [33,34]. Polyvinylpyrrolidone (PVP, *M*_w_ = 1.3 × 10^6^ g mol^−1^), poly(sodium 4-styrenesulfonate) (NaPSS, *M*_w_ = 7.0 × 10^4^ g mol^−1^) and poly(vinyl alcohol) (PVA, *M*_w_ = 6.7 × 10^4^ g mol^−1^) were purchased from Aladdin, Co., Shanghai, China. Polyacrylic acid (PAA, *M*_w_ = 4.5 × 10^5^ g mol^−1^) was purchased from Sigma-Aldrich. Organic solvents of toluene, tetrahydrofuran (THF), acetone, ethanol, DMSO and dimethylformamide (DMF) were dried prior to use.

### Fabrication of AIE/PVP microfiber sensor

Dry-spinning technology was used to fabricate AIE/PVP microfibers. Taking TPE-P/PVP microfibers as an example, TPE-P was first dissolved in THF to prepare a stock solution with a concentration of 1 mg mL^−1^. Then, 277 μL of TPE-P solution was mixed with 1.25 g PVP in 5 mL of ethanol (250 mg mL^−1^) under stirring for 2 h. Afterwards, the mixed solution as a spinning dope was transferred to a syringe with a needle diameter of 0.3 mm, which was then directly spun under a desired shear force to form microfibers immediately. In this process, the needle tip was perpendicular to the collect roller (diameter: ∼3.6 cm) with a distance of 35 cm. During the dry-spinning, the extrusion speed of spinning dope was controlled to be 2 μL min^−1^ by a syringe pump, while the paper-coated roll was autonomously rotated with a speed of ∼500 r min^−1^. The as-spun fibers were collected and then put in a drying box for subsequent study. The preparation of TPE-EP/PVP microfibers was similar to that of TPE-P/PVP.

The fabric-like AIE/PVP was also developed by dry-spinning method. It is noted that a Teflon frame (3 cm × 3 cm) was used for the collector. Fabric was obtained by rotating the Teflon frame in alternate parallel and perpendicular directions.

This dry-spinning was synchronized with the device encapsulation process to fabricate the fiber-shaped device. First, the silicone tube was inserted with tandem-connected UV LED beads to develop a silicone UV LED tube. Then, dry-spun microfibers extruded from a syringe were directly wrapped around the silicone tube to produce a fiber-shaped device.

### Fabrication of AIE/PAA nanofiber sensor

Electro-spinning method was used to fabricate a nanofiber sensor, which was conducted on the self-made NanoFiber Electrospinning Unit. A mixture of AIE molecules and PAA (weight ratio: 1/4500) were dissolved in DMF (AIE/PAA concentration: 100 mg mL^−1^) to serve as spinning dope. The above dope solution was injected through the needle of syringes under a high voltage of 12 kV (diameter of the needle: 0.26 mm; injection rate: 9 μL min^−1^). The distance between the needle tip and the collector was 20 cm. The resulting nanofibrous membrane was recovered from the collector and placed in a drying box for subsequent study.

### Materials characterizations

The morphologies of the nano-/micro-fibrous materials were characterized by SEM (HITACHI SU800), laser scanning confocal microscope (Leica TCS SP5 II) and fluorescent microscope (Nikon Eclipse Ni-U). The crystallinity of the PVA sample was characterized by wide angle X-ray diffraction (WAXD, D/Max 2550 VB PC, Rigaku). UV absorption measurements were performed on a Shimadzu UV 3600. PL spectra were collected on a PTI QM/TM. The quantum yields were measured on a HAMAMASTU fluorescence spectrometer. For demonstrating the fluorescence sensitivity of the AIE/polymer fibers, the samples were placed in a self-made moisture chamber and incubated for 1 h before characterization. The moisture (RH) was adjusted by saturated salt solutions. The RH from 11% to 95% was adjusted by saturated salt solutions of LiCl, MgCl_2_, NaBr, NaCl and KNO_3_, respectively. Fluorescent and optical images were collected on a Cannon digital camera. A MATLAB program was used to convert the optical images from RGB color space into CIE *x* and *y* chromaticity values [50].

### Theoretical calculations

All DFT calculations were performed using the Gaussian 09 software package [51]. The optimized geometries of AIE molecules and the corresponding HOMOs and LUMOs were performed at B3LYP/6–31G(d). The solvent effect was considered by the Polarizable Continuum Model [52,53]. The interaction energies between AIE molecules and polymers were calculated at B3LYP-D3(BJ)/6–31G(d, p) [54–56]. The frequency calculations were used to verify the structures with true energy minima. Only positive frequencies were contained. The interaction energy (Δ*E*) was computed via the equation of Δ*E* = *E*(AB) – *E*(A) – *E*(B) [42], where *E*(A), *E*(B) and *E*(AB) refer to the optimized ground state energies of the TPE-P, the repeat unit of polymer (PVA/PAA/PVP/NaPSS) and the complex of the TPE-P/polymer, respectively.

## Supplementary Material

nwaa135_Supplemental_File
